# Palmitoleic acid (n-7) increases white adipocytes GLUT4 content and glucose uptake in association with AMPK activation

**DOI:** 10.1186/1476-511X-13-199

**Published:** 2014-12-20

**Authors:** Andressa Bolsoni-Lopes, William T Festuccia, Patricia Chimin, Talita SM Farias, Francisco L Torres-Leal, Maysa M Cruz, Paula B Andrade, Sandro M Hirabara, Fabio B Lima, Maria Isabel C Alonso-Vale

**Affiliations:** Department of Physiology and Biophysics, Institute of Biomedical Sciences, University of Sao Paulo, Sao Paulo, Brazil; Department of Biological Sciences, Institute of Environmental Sciences, Chemical and Pharmaceutical, Federal University of Sao Paulo, 210, Sao Nicolau St., Diadema, 09913-030 Brazil; Institute of Physical Activity Sciences and Sports, Program of Post-Graduate in Human Movement Sciences, Cruzeiro do Sul University, Sao Paulo, Brazil

**Keywords:** GLUT4, AMPK, Akt, *de novo* lipogenesis, Glucose oxidation, Glucose metabolism

## Abstract

**Background:**

Palmitoleic acid was previously shown to improve glucose homeostasis by reducing hepatic glucose production and by enhancing insulin-stimulated glucose uptake in skeletal muscle. Herein we tested the hypothesis that palmitoleic acid positively modulates glucose uptake and metabolism in adipocytes.

**Methods:**

For this, both differentiated 3 T3-L1 cells treated with either palmitoleic acid (16:1n7, 200 μM) or palmitic acid (16:0, 200 μM) for 24 h and primary adipocytes from mice treated with 16:1n7 (300 mg/kg/day) or oleic acid (18:1n9, 300 mg/kg/day) by gavage for 10 days were evaluated for glucose uptake, oxidation, conversion to lactate and incorporation into fatty acids and glycerol components of TAG along with the activity and expression of lipogenic enzymes.

**Results:**

Treatment of adipocytes with palmitoleic, but not oleic (*in vivo*) or palmitic (*in vitro*) acids, increased basal and insulin-stimulated glucose uptake and GLUT4 mRNA levels and protein content. Along with uptake, palmitoleic acid enhanced glucose oxidation (aerobic glycolysis), conversion to lactate (anaerobic glycolysis) and incorporation into glycerol-TAG, but reduced *de novo* fatty acid synthesis from glucose and acetate and the activity of lipogenic enzymes glucose 6-phosphate dehydrogenase and ATP-citrate lyase. Importantly, palmitoleic acid induction of adipocyte glucose uptake and metabolism were associated with AMPK activation as evidenced by the increased protein content of phospho(p)Thr172AMPKα, but no changes in pSer473Akt and pThr308Akt. Importantly, such increase in GLUT4 content induced by 16:1n7, was prevented by pharmacological inhibition of AMPK with compound C.

**Conclusions:**

In conclusion, palmitoleic acid increases glucose uptake and the GLUT4 content in association with AMPK activation.

## Background

White adipose tissue (WAT) plays an important role in the determination of whole-body energy homeostasis through the storage and mobilization of energy in periods of surplus and demand, respectively, along with the secretion of a large variety of hormones that modulate important metabolic processes in body tissues [1, 2]. WAT also contributes to whole-body glucose homeostasis in healthy individuals responding for approximately 15% of total glucose disposal, being this hexose an important metabolic substrate for energy production and storage in adipocytes [3, 4].

Glucose uptake in adipocytes is carried out independently of insulin by specific glucose transporters (GLUTs) namely GLUT1 and GLUT5 located in the plasma membrane that display low efficiency of transport for the hexose [5]. In the presence of insulin, however, glucose uptake in adipocytes is drastically enhanced (by 10–20 fold) after translocation and fusion of intracellular vesicles containing GLUT4 to the plasma membrane [6, 7] induced by activation of the canonical insulin receptor substrate (IRS) - phosphoinositide 3-kinase (PI3K) - Akt pathway [7–9]. In addition to translocation, insulin through the very same IRS-PI3K-Akt pathway also modulates GLUT4 protein content [10, 11].

Another intracellular signaling pathway that plays an important role in the regulation of glucose uptake in adipocytes is the AMP-activated protein kinase (AMPK) [12, 13], a heterotrimeric protein that is activated by the lower ATP/AMP ratio commonly found in situations of higher energy demand. Upon its activation, AMPK promotes GLUT4 translocation to the plasma membrane and glucose uptake independently of insulin [8, 13–15]. Along with translocation, AMPK also positively modulates GLUT4 transcription and protein levels [16].

Evidences accumulated over the years have shown that fatty acids, according to the carbon chain length and number of double bounds, have the ability to affect rates of glucose uptake through the modulation of above-mentioned intracellular signaling pathways [17]. Indeed, saturated long-chain fatty acids such as palmitic (16:0) and stearic (18:0) acids were shown to impair glucose uptake [18, 19], whereas monounsaturated n-7 palmitoleic acid (16:1n7) was found to improve glucose uptake by affecting insulin responsivity [20]. More specifically to latter, palmitoleic acid, which is synthesized by the desaturation of palmitic acid (16:0) catalyzed by the stearoyl-CoA desaturase 1 (SCD-1), was shown to improve glucose homeostasis by enhancing Akt activation and plasma membrane GLUT1 and GLUT4 protein content in skeletal muscle [20–22] and by reducing hepatic esteatosis and improving insuling signaling in the liver [20, 23]. Furthermore, palmitoleic acid was also shown to protect pancreatic β-cells from the deleterious effects of palmitic acid [24, 25] and to increase lipolysis and the content of the major lipases ATGL and HSL in adipose tissue [26].

In the present study, we tested the hypothesis that, similarly to skeletal muscle, palmitoleic acid is an important modulator of glucose uptake and metabolism in adipocytes. For this, adipocytes were evaluated for glucose uptake and metabolism after treatment with palmitoleic acid. Putative mechanisms underlying palmitoleic acid actions in adipocytes were also investigated.

## Materials and methods

### Animals

All experimental protocols were approved by the Animal Ethical Care Committee of the Institute of Biomedical Sciences, University of Sao Paulo, Brazil, (#98/10/CEUA). Male 8-wk-old C57BL/6 wild type (WT) mice (from the Animal Facility of the Institute of Biomedical Sciences, University of Sao Paulo, Sao Paulo, Brazil) were kept individually in cages at 23°C on a 12:12-h light–dark cycle with food (chow pellet diet; Nuvilab CR1, Nuvital SA, Colombo, PR, Brazil) and water *ad libitum*. Mice were randomly assigned in one of the three groups that received 300 mg/kg/day of pure palmitoleic acid (16:1n7), oleic acid (18:1n9) (Sigma, St. Louis) or water by gavage [23, 26]. Instead of palmitic acid, which requires previous dissolution in organic solvent prior to administration, oleic acid was chosen as a control fatty acid for the *in vivo* experiments. Oleic acid, similarly to palmitoleic acid, does not require dissolution prior administration to mice. Gavages were carried daily between 16:00 and 17:00 h. Body weight and food intake were measured twice throughout the experiment. After 10 days, 6 h fasted mice were anesthetized with isoflurane and killed by cervical dislocation after blood collection through cardiac puncture. Blood samples were centrifuged at 1,500 rpm for 20 min at 4°C and serum was stored at -80°C. Adipose fat pads (epididymal, inguinal and retroperitoneal) were harvested, weighed and processed as described below.

### Adipocyte isolation

Adipocyte isolation was performed as previously described [27] with slight modifications. Briefly, epididymal fat pads were digested in Dulbeccos modified Eagle Medium (DMEM) supplemented with HEPES (20 mM), sodium pyruvate (2 mM), bovine serum albumin (BSA, 1%), and collagenase type II (1 mg/mL), pH 7.4 at 37°C in an orbital bath shaker. Isolated adipocytes were filtered and washed three times in the same buffer without collagenase. A small amount of adipocytes were photographed under an optical microscope (×100 magnification) using a microscope camera (Moticam 1000; Motic, Richmond, British Columbia, Canada), and mean adipocyte diameter was determined by measuring 50 cells using Motic-Images Plus 2.0 software.

### Cell culture

3 T3-L1 preadipocytes were cultured in DMEM containing 10% calf serum and penicillin/streptozotocin at 1% until confluence. After 2–3 days post-confluence, differentiation was induced by a cocktail composed of dexamethasone (1 μM), isobutylmethylxanthine (0.5 mM) and insulin (1.67 μM). After 48 h, medium was replaced by DMEM with 10% FBS containing 0.41 μM insulin [28]. Differentiated 3 T3-L1 cells (6 days after cocktail) were incubated either with vehicle (ethanol 0.05%), or palmitic acid (16:0, 200 μM) or palmitoleic acid (16:1n7, 200 μM). Because 3 T3-L1 cells are abundant in palmitoleic acid, a dose of fatty acids slightly higher than that commonly found in plasma of rodents and humans was chosen to challenge these cells *in vitro*. As evaluated by membrane integrity and DNA fragmentation (data not shown), this dose of fatty acid is not cytotoxic or deleterious to 3 T3-L1. After 18 h of treatment, cells were washed with PBS and starved from serum and insulin in the presence of fatty acids for 6 h. Treatment with palmitoleic acid for 24 h induced a significant increase in 3 T3-L1 palmitoleic acid content, without affecting cell levels of palmitic, stearic, oleic, and vaccenic acids [26]. AMPK inhibition was achieved by treatment with 6-[4-(2-Piperidin-1-ylethoxy)phenyl]-3-pyridin-4-ylpyrazolo[1,5-a]pyrimidine (Compound C, 20 μM in DMSO) to the medium containing fatty acids, for 24 h. All reagents and drugs were purchased from Sigma Chemical Company (St. Louis, MO, USA).

### 2-Deoxy-D-glucose (2-DG) uptake

Differentiated 3 T3-L1 cells (~8 × 10^5^cells/well) or primary epididymal adipocytes (10^6^ cells/mL) were incubated with or without insulin (100 or 10 nmol/L, for 3 T3-L1 and primary adipocytes, respectively) in buffer composed of (mM): 140 NaCl, 20 Hepes, 5 KCl, 2.5 MgSO4, 1 CaCl2, BSA 1% (pH 7.4), for 20 min at 37°C. Subsequently, 2-deoxy-D-[^3^H]-glucose (0.4 mmol/L, 1850 Bq/tube or well) was added and the reaction was allowed to occur for exactly 4 or 3 min, for 3 T3-L1 and primary adipocytes, respectively. The reaction was interrupted by adding 250 μl of ice-cold phloretin (0.3 mmol/L in Earle's salts, HEPES 10 mm, BSA 1% and DMSO 0.05%). 3 T3-L1 cells were washed with cold PBS, 300 μL of NaOH 50 mM was added to each well, the plate was rotated for 20 min and 250 μL was collected to measure the radioactivity (1450 LSC, Couter MicroBeta, Trilux; PerkinElmer). For epididymal primary adipocytes, 200-μl aliquots of this final mixture were layered with 200 μl of silicone oil (density of 0.963 mg/ml) in microfuge tubes and centrifuged for 10 sec at 11,000 × *g*. The cell pellet on the top of the oil layer was collected, transferred to vials containing scintillation cocktail for radioactivity measurement.

### Glucose oxidation and incorporation into fatty acids and glycerol of TAG

Differentiated 3 T3-L1 cells (~8 × 10^5^ cells/well) were incubated in Krebs/Ringer/phosphate buffer (pH 7.4) containing BSA (1%) and [U-^14^C]-glucose (2 mM, 1850 Bq/tube or well), saturated with a gas mixture of 95% O_2_ and 5% CO_2_, in the presence or absence of insulin (100 nM), for 2 h at 37°C. Prior to 2 h incubation period, each well was covered with a piece of Whatman filter paper and the plate was sealed with parafilm. Following the 2 h incubation, the filter paper was soaked with 0.1 mL of ethanolamine to trap the CO2 produced, and 0.2 mL of 8 N H_2_SO_4_ was injected into the wells to stop the reaction. After 45 min of CO_2_ trapping, the filter paper was removed and transferred to scintillation vials for radioactivity counting [29, 30]. Then, 2.5 mL of Dole's reagent (isopropanol:heptane:H_2_SO_4_, 4:1:0.25, vol/vol/vol) were added into the plates for lipid extraction. Right after the addition of heptane (1.5 mL) and distilled water (1.5 mL), the tubes were vortexed and the mixture decanted for 5 min. An aliquot of the upper phase was collected into a scintillation vial for determination of the radioactivity incorporated into TAG. For the estimation of glucose incorporation into fatty acids and glycerol of TAG, an aliquot of upper phase was transferred to a tube containing 1 mL of ethanol (95%) and 250 μL of KOH (40%) and incubated in a water bath at 60°C for 1 h. After incubation, 2 mL of HCl (3 N) and 2 ml of heptane were added, tubes were vortexed and the upper phase was transferred (1 mL) to a scintillation vial for the determination of glucose incorporation into fatty acids of TAG. Glucose incorporation into the glycerol fraction of TAG was calculated by the difference between the incorporation of [U-^14^C]-glucose into TAG and fatty acids.

### Incorporation of [1-^14^C]-acetate into fatty acids

Differentiated 3 T3-L1 cells (~8 × 10^5^cells/well) were incubated in Krebs/Ringer/phosphate buffer (pH 7.4) containing BSA (1%), glucose (2 mM) and [1-^14^C]-acetate (1 mM, 1850 Bq/tube or well) for 2 h at 37°C in a water bath. At the end of incubation, the lipids were extracted by the Dole's method for measurement of acetate incorporation into fatty acids as described above.

### Maximal activity of the enzymes involved in the *de novo*fatty acid synthesis

The activities of glucose-6-phosphate dehydrogenase (G6PDH) (EC 1.1.1.49), ATP citrate lyase (ACL) (EC 4.1.3.8) and fatty acid synthase (FAS) (EC 2.3.1.85) were analyzed. Differentiated 3 T3-L1 (~8 × 10^5^ cells/well) were homogenized in extraction buffer containing sucrose (0.25 M), EDTA (1 mM), DTT (1 mM), leupeptin (20 μg ml^-1^) and aprotinin (5 μg ml^-1^) (1:1, pH 7.4) and centrifuged at 20,000 × g at 4°C for 5 min. The fat cake free supernatant fraction was used for quantification of enzyme activities as previously described [31].

### Glucose conversion into lactate

Differentiated 3 T3-L1 (~8 × 10^5^ cells/well) were incubated in Krebs/Ringer/phosphate buffer (pH 7.4) containing BSA (1%), glucose (2 mM) for 2 h at 37°C. At the end of incubation, the medium was collected to measure the glucose converted into lactate using commercial kit (Enzymatic Lactate - Labtest Diagnóstica from Lagoa Santa, MG, Brazil).

### RNA extraction and quantitative real-time polymerase chain reaction (Real-Time qRT-PCR)

Total RNA from 3 T3-L1 cells or epididymal adipocytes was extracted with Trizol (Invitrogen Life Technologies), analyzed for quality on agarose gel and absorbance ratios of 260/280 and 260/230 nm, and reverse transcribed to cDNA using the High-Capacity cDNA kit (Applied Biosystems). Gene expression was evaluated by real-time qRT-PCR using a Rotor Gene (Qiagen) and SYBR Green as fluorescent dye with 36B4 as a housekeeping gene. PCR products were run on agarose gel to confirm the size of the fragment and specificity of amplification. Some PCR products were extracted from gel with a kit (Qiagen) and submitted to sequencing for identity confirmation. Primers used and annealing tempera0tures are presented: 36B4 (5′-3′ sense: TAAAGACTGGAGACAAGGTG; 5′-3′antisense: GTGTACTCAGTCTCCACAGA; 63°C; NM_007475) and GLUT4 (5′-3′ sense: CATTCCCTGGTTCATTGTGG; 5′-3′ antisense: GAAGACGTAAGGACCCATAGC; 60°C; NM_009204).

### Western blot analysis

For GLUT1 and GLUT 4 total protein content analysis, 3 T3-L1 cells were homogenized and processed in buffer composed in mM: 10 Tris–HCl, 1 EDTA and 250 sucrose, 7.4 pH and centrifuged at 1,000 × g for 15 minutes at 4°C [32, 33]. For the analysis of other proteins, 3 T3-L1 cells were homogenized and processed in buffer composed in mM: 50 HEPES, 40 NaCl, 50 NaF, 2 EDTA, 10 sodium pyrophosphate, 10 sodium glycerophosphate, 2 sodium orthovanadate, 1% Triton-X100, and EDTA-free protease inhibitors. Identical amounts of protein aliquots of 3 T3-L1 lysates cells were resolved on Nupage gradient gels (4-12%, Life Technologies) and transferred to nitrocellulose membranes. After blockage with 5% milk for 1 h, membranes were incubated overnight at 4°C with the following primary antibodies: GLUT 1 (#07-1401), GLUT4 (#07-1404) (Millipore, Billerica, MA, USA) or Akt (#9685S), phosphoSer473 Akt (#4060S), phosphoThr308 Akt (#4056), AMPKα (#2532) and phosphoThr172 AMPKα (#2531) (Cell Signaling, Beverly, MA, USA) or GAPDH (G9545, Sigma) in 5% milk (1:1000). After washing, membranes were subsequently incubated with appropriated peroxidase-conjugated secondary antibody (1:5000) for 1 h and developed using the ECL enhanced chemiluminescence substrate (GE Healthcare Life Sciences, Björkgatan, Uppsala). Densitometric analyses were performed using the ImageJ software (National Institutes of Health, Bethesda, MD).

### Statistical analysis

Data are expressed as mean ± SEM. Student t-Test or One-Way ANOVA (as indicated in the figure legends) followed by Tukey post-hoc test were used to compare the effects of different treatments and conditions. Analysis was performed using GraphPad Prism 5.0 software (GraphPad Software, Inc., San Diego, CA, USA). The level of significance was set at *p* ≤ 0.05.

## Results

**Figure 1:**
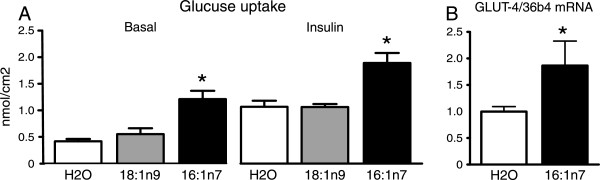
**Basal and insulin-stimulated rates of glucose uptake (Panel A), mRNA levels of glucose transporter 4 (GLUT4, Panel B) in isolated epididymal adipocytes from mice treated for 10 days by gavage with either water (H2O), oleic acid (18:1n9, 300 mg/kg/day) or palmitoleic acid (16:1n7, 300 mg/kg/day).** Results are means ± SE (*n* = 5-6/group). *P < 0.05 16:1n7 vs. all groups.

Confirming our previous study [26], palmitoleic acid administration to mice for 10 days did not affect body weight, food intake, adiposity and plasma levels of free fatty acids, insulin and glucose (data not shown). Despite the absence of changes in these variables, treatment of mice with 16:1n7, but not 18:1n9 induced a significant increase in primary epididymal adipocyte basal and insulin-stimulated glucose uptake in comparison to water treated control mice (~3-fold and 1.8-fold, respectively, Figure 1A). Importantly, such increase in glucose uptake induced by 16:1n7 was associated with a marked upregulation in adipose tissue GLUT4 mRNA levels (~86%, Figure 1B).

**Figure 2:**
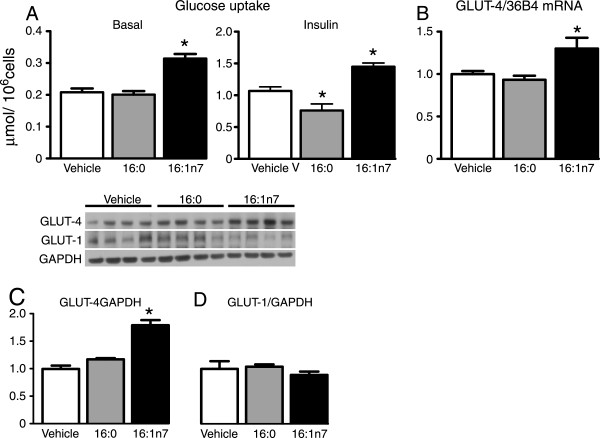
**Basal and insulin-stimulated rates of glucose uptake (Panel A), basal mRNA levels of glucose transporter 4 (GLUT4, Panel B), basal protein content of GLUT4 (Panel C) and glucose transporter 1 (GLUT1, Panel D) in differentiated 3 T3-L1 cells treated for 24 h with either vehicle, palmitic acid (16:0, 200 μM) or palmitoleic acid (16:1n7, 200 μM).** Results are means ± SE (*n* = 4-8/group). *P < 0.05 vs. all groups.

In an attempt to investigate whether palmitoleic acid enhances glucose uptake by acting directly on adipocytes and, if so, to unveil mechanisms underlying this action, we next evaluated the effects of this fatty acid on 3 T3-L1 adipocytes glucose uptake *in vitro*. In agreement with *in vivo* findings, 24 h treatment with 16:1n7 significantly increased basal and insulin-stimulated glucose uptake in 3 T3-L1 adipocytes (51% and 36%, respectively, Figure 2A), whereas treatment with 16:0 had either no effect on basal or significantly reduced insulin-stimulated glucose uptake (40%) in these cells. Similarly to *in vivo* findings, the increase in 3 T3-L1 adipocytes glucose uptake induced by 16:1n7 was associated with a significant upregulation in GLUT4 mRNA levels (34%) and protein content (78%) under basal non-stimulated conditions (Figure 2B and C). No effects of 16:0 were seen on GLUT4 expression or protein content and, none of these fatty acids significantly affected GLUT1 protein levels (Figure 2D).

**Figure 3:**
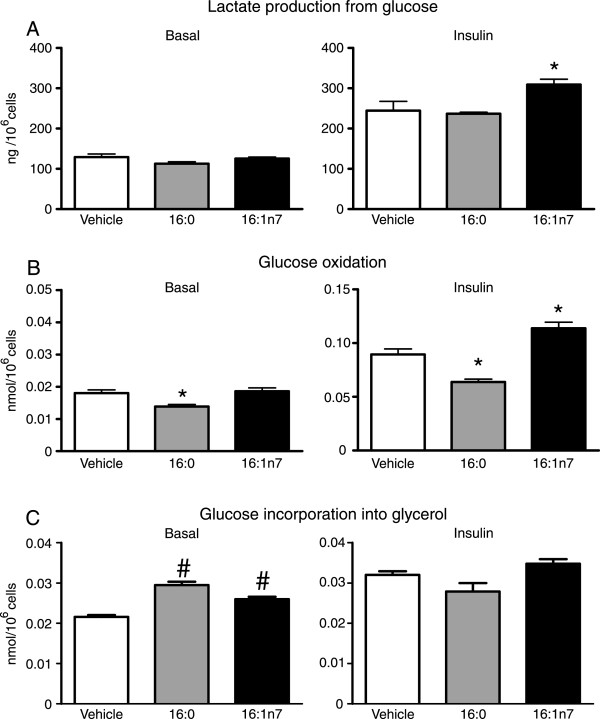
**Basal and insulin-stimulated rates of glucose conversion into lactate (Panel A), glucose oxidation (Panel B) and glucose incorporation into glycerol fraction of triacylglycerol (Panel C), in differentiated 3 T3-L1 cells treated for 24 h with either vehicle, palmitic acid (16:0, 200 μM) or palmitoleic acid (16:1n7, 200 μM). Results are means ± SE (** ***n*** **= 6-10/group).** *P < 0.05 vs. all groups and ^#^P < 0.05 vs. vehicle.

In face of these changes in glucose uptake, we next investigated whether 16:1n7 also affects glucose metabolism in 3 T3-L1 adipocytes. As depicted in Figure 3A and B, palmitoleic acid significantly increased insulin-stimulated glucose conversion into lactate and oxidation to CO2 in comparison to vehicle treated cells (~29% and 27%, respectively). Treatment with 16:0, on the other hand, significantly reduced glucose oxidation at both basal and insulin stimulated conditions, without however, affecting lactate production from glucose. Despite having opposite actions on glycolysis, treatment with both 16:1n7 and 16:0 significantly increased the generation of glycerol 3-phosphate from glucose (~37% and 21%, respectively), as evidenced by the higher rates of glucose incorporation in the glycerol fraction of TAG under basal, but not insulin stimulated conditions in comparison to vehicle treated cells (Figure 3C).

**Figure 4:**
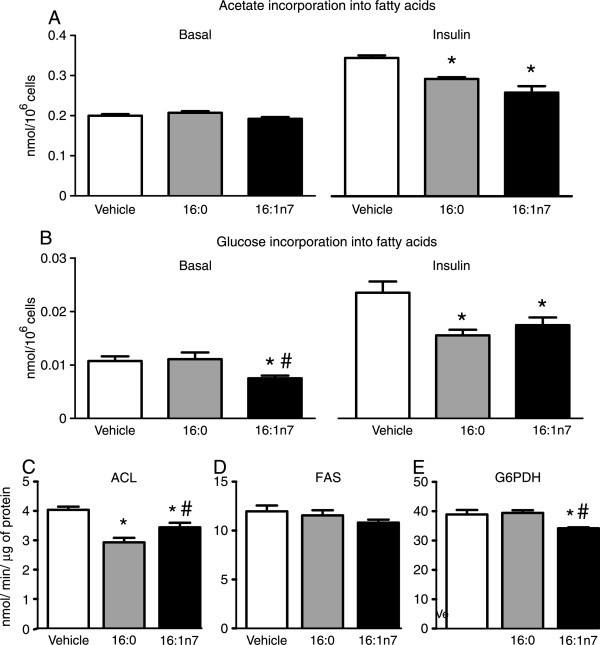
**Basal and insulin-stimulated rates of acetate (Panel A) and glucose (Panel B) incorporation in fatty acids of triacylglycerol and maximal activities of ATP citrate lyase (ACL, Panel C), fatty acid synthase (FAS, Panel D) and glucose-6-phosphate dehydrogenase (G6PDH, Panel E), in differentiated 3 T3-L1 cells treated for 24 h with either vehicle, palmitic acid (16:0, 200 μM) or palmitoleic acid (16:1n7, 200 μM).** Results are means ± SE (*n* = 6-8/group). *P < 0.05 vs. vehicle and ^#^P < 0.05 vs. 16:0.

In contrast to the synthesis of glycerol from TAG, treatment with both 16:1n7 and 16:0 decreased insulin-stimulated *de novo* lipogenesis from all sources and from glucose as estimated by acetate and glucose incorporation into fatty acids of TAG, respectively (Figure 4A and B). Treatment with 16:1n7, but not 16:0 also reduced fatty acid synthesis from glucose at basal non-stimulated conditions. Corroborating with the reduction in lipogenesis, the maximal activity of ACL, an enzyme that generates acetyl-CoA in the cytosol for fatty acid synthesis, was significantly reduced by treatment with both 16:1n7 and 16:0 (Figure 4C). Indeed, 16:1n7 alone reduced the activity of G6PDH that catalyzes the generation of NAPDH required for lipogenesis. No effects of 16:1n7 and 16:0 were seen on maximal FAS activity (Figure 4D-E).

**Figure 5:**
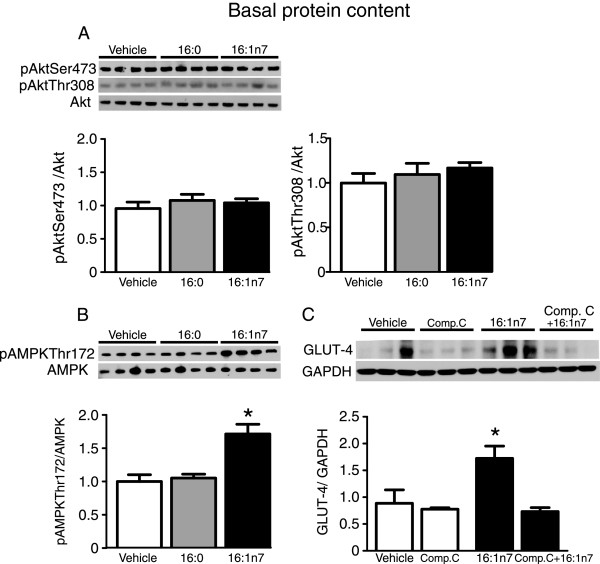
**Basal protein content of phospho(p) Ser473 Akt and pThr308Akt (Panel A) and pThr172 AMP-activated protein kinase alpha (Panel B) in differentiated 3 T3-L1 cells treated for 24 h with either vehicle, palmitic acid (16:0, 200 μM) or palmitoleic acid (16:1n7, 200 μM).** Basal protein content of glucose transporter 4 (GLUT4, Panel C) in differentiated 3 T3-L1 cells treated for 24 h with either vehicle, palmitoleic acid (16:1n7, 200 μM), Compound C (Comp. C, 20 μM) or palmitoleic acid associated with Compound C (Comp. C + 16:1n7). Results are means ± SE (*n* = 3-4/group). *P < 0.05 16:1n7 vs. all groups.

In an attempt to unveil the mechanisms underlying the above described 16:1n7 actions in adipocytes, we next investigated the effects of this fatty acid on the activation status of major intracellular signaling proteins, namely Akt and AMPK, previously implicated in the regulation of glucose uptake and metabolism. As illustrated in the Figure 5A, treatment with either with 16:1n7 or 16:0 did not affect Akt activation in 3 T3-L1 adipocytes as evidenced by the absence of changes in the content of phospho(p) Ser473 Akt and pThr308 Akt at non-stimulated conditions. In contrast to Akt, however, treatment with 16:1n7, but not 16:0, significantly increased AMPK activity as evidenced by the higher content of pThr172AMPKα (Figure 5B). Accordingly with a likely involvement of AMPK activation as a mediator of the increase in glucose uptake induced by 16:1n7, pharmacological inhibition of this kinase with compound C completely blocked the increased in GLUT4 protein content induced by treatment with 16:1n7 (Figure 5C).

## Discussion

Herein we tested the hypothesis that palmitoleic acid positively modulates glucose uptake and metabolism in white adipocytes. Through a combination of *in vivo* and *in vitro* experiments, we have found that palmitoleic acid increases adipocytes glucose uptake and the expression and content of the major glucose transporter GLUT4. Along with uptake, palmitoleic acid enhances adipocyte glucose metabolism through energy-producing instead of energy-storing pathways as evidenced by the increased rates of aerobic and anaerobic glycolysis and inhibition of *de novo* lipogenesis. Our findings also indicate that at least part of these palmitoleic acid actions seem to be due to an increase in AMPK activity induced by this fatty acid in adipocytes. Altogether our results suggest that palmitoleic acid is an important positive modulator of adipocytes glucose metabolism through energy producing pathways.

The seminal discovery that palmitoleic acid, a fatty acid endogenously synthesized by the desaturation of palmitic acid (16:0) catalyzed by SCD-1, acts as an signaling molecule that improves glucose homeostasis by enhancing glucose disposal and insulin sensitivity in skeletal muscle and by reducing glucose production and lipid deposition in liver [20, 23], motivated us to investigate possible actions of this fatty acid on adipocytes glucose uptake and metabolism. Similarly to skeletal muscle, we demonstrate herein that palmitoleic acid is a positive modulator of glucose uptake and GLUT4 expression and content in white adipocytes. Importantly, considering our findings that palmitoleic acid mainly upregulates GLUT4 content and glucose uptake at basal non-stimulated condition, it is plausible to envisage that such effect may be also responsible for the enhanced glucose uptake seem upon insulin stimulation in cells treated with this fatty acid. In agreement with this notion, palmitoleic acid induction of glucose uptake in adipocytes occurred without major changes on pAkt content, which strongly indicates that other intracellular signaling pathway than the canonical insulin regulated IRS-PI3K-Akt is involved in these actions. These findings, which are in contrast to previous studies showing that palmitoleic acid increases pAkt and IRS-1 and 2 contents in liver and skeletal muscle [20–22], also indicates that palmitoleic acid increases glucose uptake in adipocytes through different mechanisms of action. In this sense, we have found that palmitoleic acid induces a marked activation of AMPK in adipocytes, a heterotrimeric kinase activated by an impairment in the ratio ATP/AMP in situations of increased energy demand [12, 13]. In addition to the activation of GLUT4 translocation into the membrane independently of insulin [13], AMPK is also a positive modulator of GLUT4 expression and content, such effects that seem to involve the phosphorylation and activation of peroxisome proliferator-activated receptor gamma coactivator 1α (PGC1α) and histone deacetylase 5 (HDAC5) [34, 35]. Further supporting our hypothesis that palmitoleic acid increases glucose uptake and GLUT4 content in adipocytes by activating AMPK, pharmacological inhibition of this kinase with Compound C completely blocked the increased in GLUT4 content induced by palmitoleic acid. Although Compound C has been widely used as a cell-permeable AMPK inhibitor in *in vitro* and *in vivo* experiments including those conducted in adipocytes, a recent study has found that this drug has a poor specificity targeting other proteins than AMPK [10, 14, 36]. Taken this limitation into account, altogether our findings suggest that palmitoleic acid similarly to exercise, adiponectin, 5-amino-1-β-D-ribofuranosyl-imidazole-4-carboxamide (AICAR), pachymic acid among others, is an important activator of glucose uptake in association with AMPK activation [10, 14, 16, 37].

In addition to uptake, palmitoleic acid significantly modulated glucose flux through its major metabolic pathways in adipocytes. At basal, non-stimulated conditions, both palmitoleic and palmitic acids enhanced glucose conversion into glycerol 3-phosphate, the carbon backbone for fatty acid esterification and TAG synthesis. Although of unknown underlying mechanism, these findings suggest that adipocytes adjust rates of glycerol 3-phosphate generation according to availability of fatty acid, but independently of their identity. More specifically to palmitoleic acid, however, we have shown in previous study [26] that this fatty acid increases glycerol 3-phosphate generation concomitantly to lipolysis, thus promoting an elevation in the recycling of lipolysis-derived fatty acid back to TAG. This process defined as TAG-fatty acid cycling has been shown to enhance cell energy expenditure and the sensitivity of neuro/hormonal control [38–40]. The absence of changes in both glucose oxidation and conversion to lactate at basal, non-stimulated conditions indicate, however, that other substrates than glucose, are being metabolized to account for the increased demand of energy promoted by the enhanced TAG-fatty acid cycle.

Upon insulin stimulation, however, palmitoleic acid enhanced glucose metabolism to both CO2 and lactate and significantly reduced *de novo* fatty synthesis as evidenced by the impaired incorporation of acetate (total) and glucose into fatty acids and the activities of G6PDH and ACL enzymes. These findings suggest that in the presence of insulin, palmitoleic acid favors the activation of energy-producing instead of energy-storing metabolic pathways. Similarly to glucose uptake, AMPK may account for at least part of these palmitoleic acid actions, since activation of this kinase has been shown not only to increase glucose flux trough glycolysis leading to higher rates of hexose oxidation [37, 41], but also to inhibit *de novo* lipogenesis by phosphorylating and inactivating acetyl-CoA-carboxylase (ACC), thus reducing the conversion of citosolic acetyl-CoA to malonyl-CoA, a precursor for fatty acid synthesis [13, 42]. Importantly, our findings that palmitic acid also reduces *de novo* lipogenesis in adipocytes not only suggest that this process, similarly to glycerol 3-phosphate generation, is modulated by cell fatty acid availability, but also that AMPK may not be the only mechanism underlying these effects of fatty acids, since in contrast to palmitoleic acid, palmitic acid did not affect the activity of this kinase.

Although the mechanisms by which palmitoleic acid activates AMPK in adipocytes were not elucidated here, our recent findings that this fatty acid enhances lipolysis and TAG-fatty cycle [26] suggest a relationship between these processes. Corroborating this hypothesis, a previous study has found that lipolysis activation leads to a significant increase in AMPK activity that seems to be due to a reduction of 25% in cellular ATP levels associated to an increase in the activity of acyl-CoA synthase and fatty acid reesterification into TAG [43]. Further studies, however, are required to test this hypothesis and to define the mechanisms by which palmitoleic acid activates AMPK in adipocytes.

In summary, we have presented evidence that palmitoleic acid, in addition to its effects on liver and skeletal muscle, is a key regulator of glucose uptake and metabolism in white adipose tissue. Our data indicate that palmitoleic acid is an important positive modulator of glucose uptake and protein and mRNA content of GLUT4 favoring the cellular glucose utilization towards energy producing pathways, such effects that seem to be associated, at least in part, with AMPK activation. Further studies, however, are required to elucidate the mechanisms through which palmitoleic acid activates AMPK and whether adipose tissue contributes to the improvement in whole body glucose homeostasis induced by this fatty acid.
